# Analysis of dose measurement other than the radiation protection during the radiographic examination

**DOI:** 10.1186/2193-1801-3-250

**Published:** 2014-05-17

**Authors:** Ki-Youl Kim, Jae-Hwan Cho, Hae-Kag Lee

**Affiliations:** Department of Radiology, Kyung Hee University Hospital at Gang-dong, Seoul, Republic of Korea; Department of International Radiological Science, Hallym University of Graduate Studies, Seoul, Republic of Korea; Department of Computer Engineering, Soonchunhyang University, Asan, Chungnam 336-745 Republic of Korea

**Keywords:** Exposure dose, General shooting, Scattered rays

## Abstract

**Objectives:**

The study measured the dose on body regions that were not shielded to protect from radiation exposure during the general procedure, with the goal of providing basic radiation dose data for radiological technologists who perform the radiographic examination.

**Materials and methods:**

Shooting parts with the phantom were similar to human tissues using general shooting equipment in the general examination room. The scattered rays were measured with the ion chamber. The hand received the highest average radiation dose and the kidney the lowest. The same pattern was evident for the average equivalent dose. The available daily shooting was highest in the anterior/posterior skull, followed by the posterior/anterior chest, abdomen, anterior/posterior spine and extremities.

**Results:**

The daily available numbers for the eye were lower than other body regions (6-times, 4-times, 26-times, 3-times and 121-times) and the numbers on the foot were higher than for other regions (73-times, 48-times, 263-times, 39-times and 702-times).

**Conclusions:**

Radiation should be thoroughly blocked by the apron to protect the radiological technologist from the radiation exposure, the proper distance from the irradiation source should be maintained exposure is inevitable and the exposure dose and working environment shall be regularly assessed to ensure minimal exposure dose of the radiological technologist in accordance with the International Commission on Radiological Protection recommendation.

## Introduction

Radiation-based interventional procedures and diagnostic examinations have increased in use, which has increased the exposure dose of medical staff, radiation officials and patients (Hwang et al. 2011). In Korea, diagnostic X-ray examinations have become increasingly popular; natural and artificial radiation exposure accounts for 81% and 19% of the total exposure, respectively. Radiation exposure associated with diagnostic radiology accounts for about 17% of the total radiation exposure and 92% of the the artificial radiation exposure. A management system at the national level would be helpful in decreasing the exposure of patients and in the assessment of the radiological dose for the patients (Korea Institute of Nuclear Safety 2005).

The medically-based radiological dose limit for patients has been established internationally (ICRP Publication 60 1991). The radiological dose received by patients during X-ray examination depends on the body region being examined and the policy of the medical institution/country performing the examination. The individual radiological dose is based on the type of radiographic examination and the institution/country (Lee et al. 2009). International organizations including the International Atomic Energy Agency (IAEA) and the International Commission on Radiological Protection (ICRP) proposed a recommended dose and reference level for medical diagnostic exposure of staff and patients ((IAEA 1996); (ICRP 2002)). The ICRP 60 recommended individual exposure dose for radiological personnel is <50 mSv annually or 100 mSv every 5 years (Health and Welfare Enforcement Ordinance 2001). To achieve the 5-year exposure level, exposure should not exceed 5 mSv/quarter or 20 mSv/year. A radiological technologist performs the examination while wearing personal protection equipment. However, the existing equipment only covers the abdomen and genitals. General shooting mainly consists of the extremities, chest, skull, abdomen and spine. This study measured the dose on the body parts not protected from radiation exposure during the general shooting, with the goal of providing basic data concerning radiation exposure.

## Research method

### Research equipment

**Figure 1:**
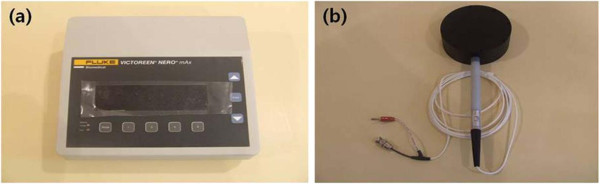
**The Model 8000 X-ray test device was used to measure the dose (a) and the external scatter ion chamber was used to measure the secondary scattered rays (b).**

**Figure 2:**
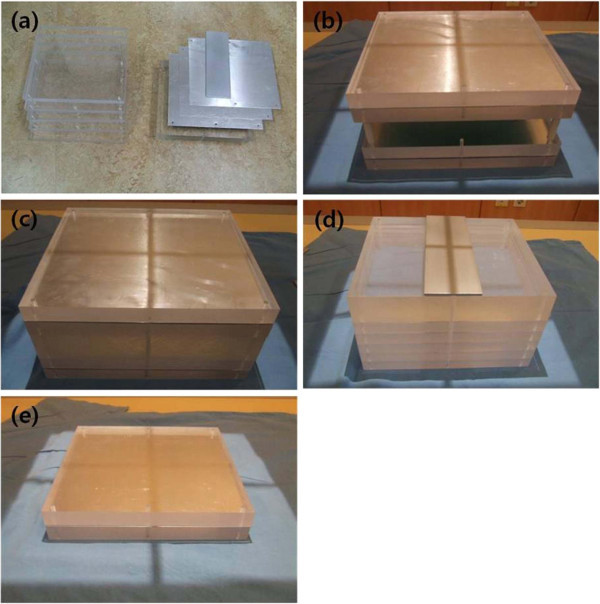
**The phantom used in the experiment was the diagnostic X-ray phantom (a).** The phantom was assembled with four types: chest (b), skull (c), abdomen and lumbar spine (d) and extremity (e).

The DIGITAL DIAGNOST X-ray equipment was used for the experiment and the X-ray Test Device (Victoreen® NERO® mAx Model 8000, USA) was used for the dose measurement. The 400 cm^3^ external scatter ion chamber of Victoreen® NERO® mAx Model 8000 X-ray device was used to measure the secondary scattered rays (Figure 1). The phantom used in the experiment was the Diagnostic X-Ray Phantom Model 76–2 Series. The phantom was assembled with four types: chest, skull, abdomen and lumbar spine and extremity (Figure 2). The apron used in the experiment was the 0.35 mm front-type Apron Pb.

### Research method

**Figure 3:**
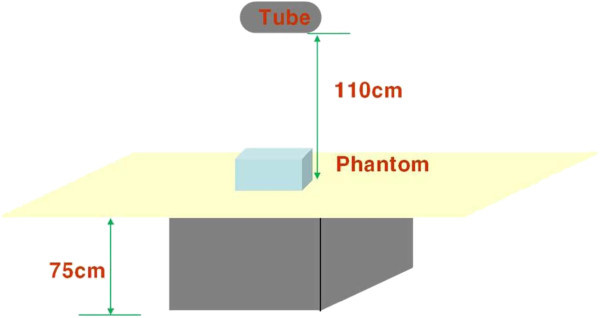
**The distance between the X-ray equipment stand and the floor was fixed to 75 cm and the distance between the focus and the table was 110 cm.**

**Figure 4:**
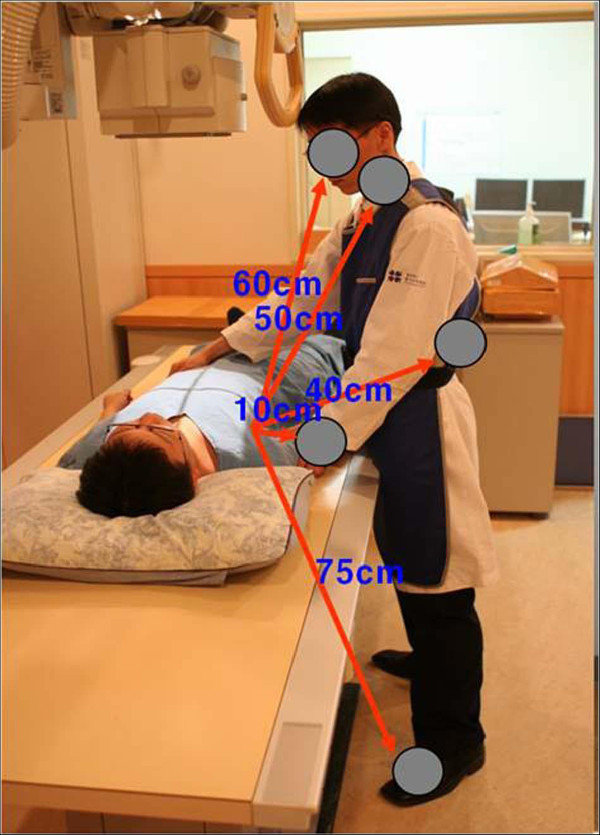
**Site of dose measurement are hand, foot, thyroid, eyeball and kidney.** First, the hand was placed 10 cm from the collimation edge to the center. Second, the foot was placed 60 cm below the table from the collimation edge to the center. Third, the thyroid was placed 50 cm above the table from the collimation edge to the center. Fourth, the eyeball was placed 60 cm from the collimation edge to the center. Fifth, the external scatter ion chamber was placed 40 cm behind the apron from the collimation edge to the center while wearing the apron because the kidney was placed behind the back.

The distance between the X-ray equipment stand and the floor was fix at 75 cm and the distance between the focus and the table was set to 110 cm. The chest phantom was placed on the X-ray equipment table, collimated with a 14 × 17 cm^2^ size area and precisely located on the beam center (Figure 3). The hand, foot, thyroid, eyeball and kidney – five body regions not covered during the chest examination of a patient – were selected and the external scatter ion chambers were placed to measure the scattered X-rays with exposure conditions of the chest (60 peak kilovoltage (kVp), 10 milliampere second (mAs)) typically used by the hospital. The secondary scattered rays were measured by the radiated dose. First, the external scatter ion chamber was placed 10 cm from the edge of the collimation to the center and exposed 20 times to measure the secondary scattered rays. The average for the hand was calculated. Secondly, the external scatter ion chamber was placed 60 cm below the table from the collimation edge to the center and exposed 20 times to measure the secondary scattered rays. The average for the foot was calculated. Third, the external scatter ion chamber was placed 50 cm above the table from the edge of the collimation to the center and exposed five times to measure the secondary scattered rays. The average for the thyroid was calculated. Fourth, the external scatter ion chamber was placed 60 cm above from the collimation edge to the center and exposed for 20 times to measure the secondary scattered rays. The average for the eyeball was calculated. Fifth, the external scatter ion chamber was placed 40 cm behind the apron from the collimation edge to the center while wearing the apron because the kidney was placed behind the back and exposed for 20 times to measure the secondary scattered rays. The average for the kidney was calculated (Figure 4). Under the same conditions, the skull phantom (exposure condition: 70 kVp, 25 mAs), abdomen and lumbar phantom (exposure condition for the abdomen: 77 kVp, 32 mAs, lumbar spine: 77 kVp, 40 mAs) and extremity phantom (exposure condition: 50 kVp, 5 mAs) were placed instead of the chest phantom, the external scatter ion chamber was placed on the same position and exposed for 20 times to measure the secondary scattered rays and the average was calculated. The values were converted to the equivalent dose based on the measured irradiation dose as in Eq. (1):

1

where, 1 Gy = 100 rad and the weighting factor on the radiation is multiplied (because of the photon for the irradiation dose) to calculate the equivalent dose, as shown in Eq. 2:2

Also, the daily number of shooting available for each part was calculated based on the ICRP 60 recommendation^(3)^ with the measured result.

The dose comparison for each part during the examination was analyzed and compared by the ANOVA test using SPSS 18.0 software (SPSS win 18.0, Chicago, USA) and a p-value < 0.05 indicated significance.

## Results

The average irradiation dose for a single shooting (Table 1) was highest for the hand (0.82 ± 0.23 mR), followed by the eyeball (0.52 ± 0.68 mR) in the extremity test. The kidney was the lowest (0.08 ± 0.02 mR) (p < 0.05). The chest anteroposterior (AP) showed that the hand was the highest (5.33 ± 1.28 mR), followed by the thyroid (2.72 ± 0.52 mR), while the kidney was the lowest (0.06 ± 0.01 mR) (p < 0.05). The skull AP showed that the hand was the highest (16.52 ± 2.35 mR), followed by the thyroid (13.34 ± 2.01 mR), while the kidney was the lowest (0.14 ± 0.06 mR) (p < 0.05). The abdomen spine examination showed that the hand was the highest (33.82 ± 6.52 mR), followed by the eyeball (17.58 ± 2.98 mR) in the extremity test, while the kidney was the lowest (0.48 ± 0.15 mR) (p < 0.05). The spine AP examination showed that the hand was the highest (40.32 ± 7.65 mR), followed by the eyeball (22.12 ± 3.23 mR) in the extremity test, while the kidney was measured the lowest (0.52 ± 0.09 mR) (p < 0.05) (Table 1). In the extremity examination (Table 2), the average equivalent dose for a single shooting showed that the hand was the highest (7.19 ± 2.01 uSv) and the kidney was the lowest (0.70 ± 0.17 uSv) (p < 0.05). In the chest AP examination, the hand was the highest (46.74 ± 11.22 uSv) and the kidney was the lowest (0.52 ± 0.08 uSv) (p < 0.05). In the skull AP examination, the hand was the highest (144.88 ± 20.60 uSv) and the kidney was the lowest (1.22 ± 0.82 uSv) (p < 0.05). In the abdomen spine examination, the hand was the highest (296.60 ± 87.18 uSv) and the kidney was the lowest (4.20 ± 1.31 uSv) (p < 0.05). In the spine AP examination, the hand was the highest (353.60 ± 67.09 uSv) and the kidney was the lowest (4.56 ± 0.78 uSv) (p < 0.05).

**Table 1:** **The average irradiation dose for a single shooting**

Measurement sites	Hand	Kidney	Thyroid	Eyeball	Foot	P
	(10 cm)	(40 cm)	(50 cm)	(60 cm)	(75 cm)	
Extremity	0.82 ± 0.23	0.08 ± 0.02	0.42 ± 0.13	0.52 ± 0.18	0.31 ± 0.17	0.025
Chest anteroposterior	5.33 ± 1.28	0.06 ± 0.01	2.72 ± 0.52	2.41 ± 0.65	0.82 ± 0.21	0.040
Skull anteroposterior	16.52 ± 2.35	0.14 ± 0.06	13.34 ± 2.01	10.34 ± 1.65	2.91 ± 0.82	0.045
Abdomen Supine	33.82 ± 6.52	0.48 ± 0.15	16.66 ± 2.56	17.58 ± 2.98	4.36 ± 1.25	0.040
Spine anteroposterior	40.32 ± 7.65	0.52 ± 0.09	21.05 ± 3.02	22.12 ± 3.23	5.46 ± 1.67	0.023

Unit: mR.

**Table 2:** **The average equivalent irradiation dose for a single shooting**

Measurement sites	Hand	Kidney	Thyroid	Eyeball	Foot	P
	(10 cm)	(40 cm)	(50 cm)	(60 cm)	(75 cm)	
Extremity	7.19 ± 2.01	0.70 ± 0.17	3.68 ± 1.14	4.56 ± 1.57	2.71 ± 1.49	0.025
Chest anteroposterior	46.74 ± 11.22	0.52 ± 0.08	23.85 ± 4.56	21.13 ± 5.70	7.19 ± 1.84	0.040
Skull anteroposterior	144.88 ± 20.60	1.22 ± 0.82	116.99 ± 17.62	90.68 ± 14.47	25.52 ± 7.19	0.045
Abdomen Supine	296.60 ± 87.18	4.20 ± 1.31	146.10 ± 22.45	154.17 ± 26.13	38.23 ± 10.96	0.040
Spine anteroposterior	353.60 ± 67.09	4.56 ± 0.78	184.60 ± 26.48	193.99 ± 28.32	47.88 ± 14.64	0.023

Unit: μSv.

**Figure 5:**
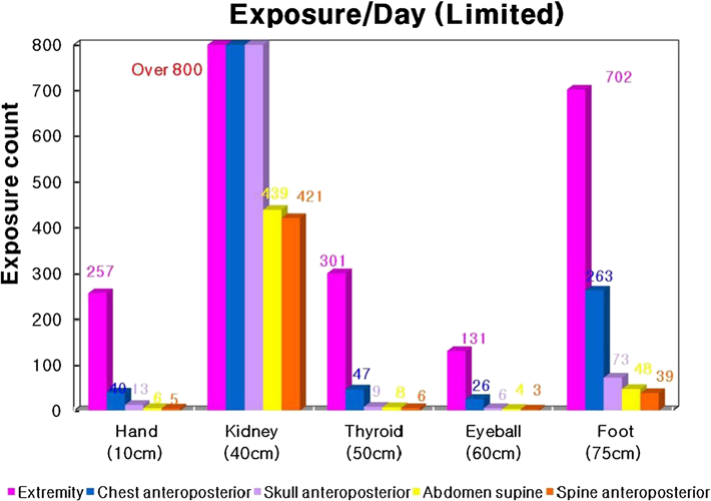
**Daily shooting available for each part during the examination based on the ICRP 60 recommendation.** 1. Research paper published in 2005 by the Ministry of Science and Technology. 2. Annual summary (2008) of the individual exposure dose for medical staff.

The available shooting a day is the highest in the skull AP, followed by the chest PA, abdomen, spine AP and extremity and the daily available numbers on the eye are lower than other parts of 6, 4, 26, 3 and 121 times and the numbers on the foot are higher than other parts of 73, 48, 263, 39 and 702 times (Figure 5).

## Discussion

At the time of the establishment of the first safety management system for the diagnostic radiation exposure of field workers by the Ministry of Health and Welfare of Korea, the number of affected personnel was 12,652. However, by 2008, the number of workers had increased almost 4-times to 47,823. The increase reflected the upgrade of the medical welfare and the greater interest in healthcare, which prompted more diagnostic radiology examinations. This increase is expected to continue (Korea Food & Drug Administration 2008). Radiation exposure has increased along with the increase in the number of examinations. The historical ratio between natural radiation and artificial radiation (85:15) has markedly changed, and is now 1:1. The increased frequency of radiographic examinations has been documented ((Brenner & Hall 2007); (Tubiana et al. 2009); (International Agency for Research on Cancer 2000); (Archer and Wagner 2000)). While the exposure dose of patients has been amply researched, only a handful of studies have addressed the exposure dose for radiological technologists.

The current study measured the dose on regions of the body that are not typically shielded by radiation protection equipment for the radiological technologist during general shooting. The hand was measured highest (7.19 ± 2.01 uSv) for the extremity test, 46.74 ± 11.22 uSv for the chest AP, 144.88 ± 20.60 uSv for the skull AP, 296.60 ± 87.18 uSv for the abdomen supine and 353.60 ± 67.09 uSv for the spine AP. Even though the daily dose was low, the accumulation over several years may not be ignored.

In a 1982 report, the United Nations Scientific Committee on the Effects of Atomic Radiation judged that there was no dangerous cause of death, except cancer under low-dose radiation (United Nations Scientific Committee on the Effects of Atomic Radiation 1982). However, a subsequent series of animal tests indicated a more substantial role of radiation. This idea was bolstered by recent data from survivors of the atomic bomb explosions in Japan near the conclusion of World War II. However, a report published in 2010 stated that the cataract was related to the low-dose radiation exposure and that radiation exposure should be restricted to research for the cardiovascular diseases (United Nations Scientific Committee on the Effects of Atomic Radiation 2010). Also, the Biological Effects of Ionizing Radiation VII report summary showed that the fetus under the radiation more than 10 mGy in the uterus increased the childhood cancer and indicated the excess risk of 6% per Gy (Doll & Wakeford 1997). Sodickson et al. (Sodickson et al. 2009) reported that 33% of patients who received more than five CT examinations in 22 years and 15% of 31,462 patients were under the effective dose higher than 100 mSv. The expected rate of cancer occurrence was 0.7%. (Brenner et al. 2001) estimated that the lifetime cancer mortality risk was 0.18% for a single abdominal computed tomography (CT) for a 1-year old infant and the number of cancer-related deaths could reache 5 million cases from 600,000 abdominal and head CT for a year.

Studies reporting an increased cancer prevalence rate and the relationship of low-dose radiation exposure have focused on the increase in the radiation exposure for the medical use. The exposure dose for radiological technologists has rapidly increased. The effects of repeated low-dose exposure require study.

## Conclusion

Radiation should be thoroughly blocked by the apron to protect the radiological technologist from radiation exposure. Furthermore, a technologist-patient distance judged to be safe should be maintained if exposure is inevitable. Finally, the exposure dose and working environment should be regularly assessed to help decrease the exposure dose of the radiological technologist in accordance with the ICRP recommendation.
